# The role of biomechanical factors in models of intervertebral disc degeneration across multiple length scales

**DOI:** 10.1063/5.0137698

**Published:** 2023-05-08

**Authors:** Daniela Lazaro-Pacheco, Mina Mohseni, Samuel Rudd, Justin Cooper-White, Timothy Patrick Holsgrove

**Affiliations:** 1 Department of Engineering, University of Exeter, Harrison Building, Streatham Campus, North Park Road, Exeter EX4 4QF, United Kingdom; 2 School of Chemical Engineering, The University of Queensland, St. Lucia QLD 4072, Australia; 3 The UQ Centre in Stem Cell Ageing and Regenerative Engineering (StemCARE), Australian Institute for Bioengineering and Nanotechnology, The University of Queensland, St. Lucia QLD 4072, Australia

## Abstract

Low back pain is the leading cause of disability, producing a substantial socio-economic burden on healthcare systems worldwide. Intervertebral disc (IVD) degeneration is a primary cause of lower back pain, and while regenerative therapies aimed at full functional recovery of the disc have been developed in recent years, no commercially available, approved devices or therapies for the regeneration of the IVD currently exist. In the development of these new approaches, numerous models for mechanical stimulation and preclinical assessment, including *in vitro* cell studies using microfluidics, *ex vivo* organ studies coupled with bioreactors and mechanical testing rigs, and *in vivo* testing in a variety of large and small animals, have emerged. These approaches have provided different capabilities, certainly improving the preclinical evaluation of these regenerative therapies, but challenges within the research environment, and compromises relating to non-representative mechanical stimulation and unrealistic test conditions, remain to be resolved. In this review, insights into the ideal characteristics of a disc model for the testing of IVD regenerative approaches are first assessed. Key learnings from *in vivo*, *ex vivo*, and *in vitro* IVD models under mechanical loading stimulation to date are presented alongside the merits and limitations of each model based on the physiological resemblance to the human IVD environment (biological and mechanical) as well as the possible feedback and output measurements for each approach. When moving from simplified *in vitro* models to *ex vivo* and *in vivo* approaches, the complexity increases resulting in less controllable models but providing a better representation of the physiological environment. Although cost, time, and ethical constraints are dependent on each approach, they escalate with the model complexity. These constraints are discussed and weighted as part of the characteristics of each model.

## I. INTRODUCTION

Low back pain (LBP) is a common condition that affects people of all ages^1^ and is a chronic cause of disability and early retirement across the globe, affecting most people over the age of 40.^1^ LBP has maintained the number one position as the leading cause of disability for the last 30 years, suggestive of slow progress in addressing the causes of this condition.^2^ While often overlooked, the direct and indirect cost of LBP is similar to the expenses incurred by cardiovascular disease, cancer, mental health, and autoimmune diseases.^3^ The estimated annual cost of management and alleviation of LBP in the United States alone is $90 billion.^4^ In Europe, the cost of musculoskeletal conditions equates to 2% of GDP affecting the wellbeing of 44 × 10^6^ European workers.^5^ Disability and low back pain related costs are expected to increase in coming decades with ageing populations globally, emphasizing the need for better therapies for the effective management and treatment of LBP, and ideally, full functional recovery of the lower back.

LBP is a symptom caused by a multitude of factors and comorbidities, with physical degeneration of the intervertebral disc (IVD) being acknowledged as a major cause.^6–8^ The IVD is a flexible cartilaginous joint between vertebrae in the spine that facilitates multidirectional movement and shock absorption during motion of the spine.^9^

When patients do not benefit from non-surgical treatments and present severe and persistent neurological symptoms, lumbar decompression surgery may be recommended.^10^ Spinal fusion mechanically joins two vertebrae together, which may alleviate the pain but imposes significant restriction and changes in biomechanical forces across the “joint,” in many cases resulting in further degeneration of the adjacent discs and vertebrae.^11^ Disc replacement with permanent implants is an alternative to spinal fusion,^12^ allowing for partial restoration of the joints original movement and functionality. However, depending on the intrinsic mechanics and wear resistance of the implant, absorption and redistribution of compressive forces can be suboptimal. Along with the generation of wear particles, resulting in osteolysis, these implants can induce stress shielding, and may dislodge and loosen as a result.^13^

The limited efficacy of current implants used for total disc replacements has spurred research into tissue engineering approaches, which aim to provide a biomaterial scaffold for new tissue generation and remodeling. These approaches have been widely discussed in recent years, highlighting the disadvantages of current therapies for IVD degeneration.^8,14–16^ A lack of a comprehensive understanding of the physiology of the degenerated disc, as well as limitations in testing new devices under physiological conditions, which fully mimic the mechanics of the spine, present significant challenges in development of efficient and translatable tissue engineered therapies for degenerated IVDs. In this regard, models that replicate real-life physiological conditions of IVDs are of particular importance. This type of model would facilitate the investigation and discovery of the complex cascades involved in IVD degeneration as well as the assessment and tailoring of newly developed devices and/or therapies in a more efficient and fit-for-purpose way. The capability to complete efficient, effective, and reliable testing of regenerative IVD treatments will provide a significant step toward the successful translation of devices and therapies to the clinical and commercial setting.

### A. Mechano-biochemical interactions involved in Degenerative Disc Disease (DDD)

**FIG. 1.**
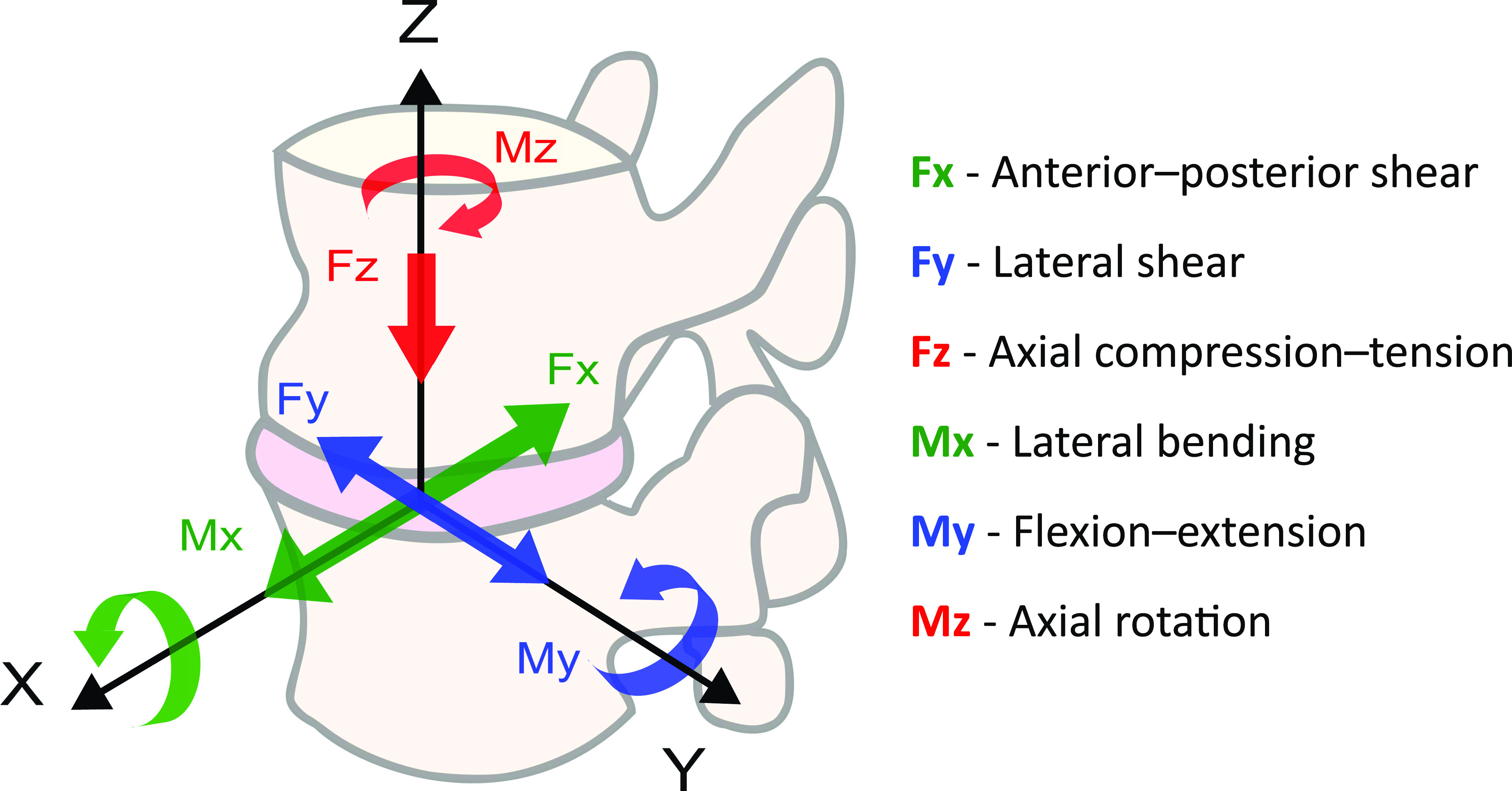
Forces and torques acting on the intervertebral disc.

**FIG. 2.**
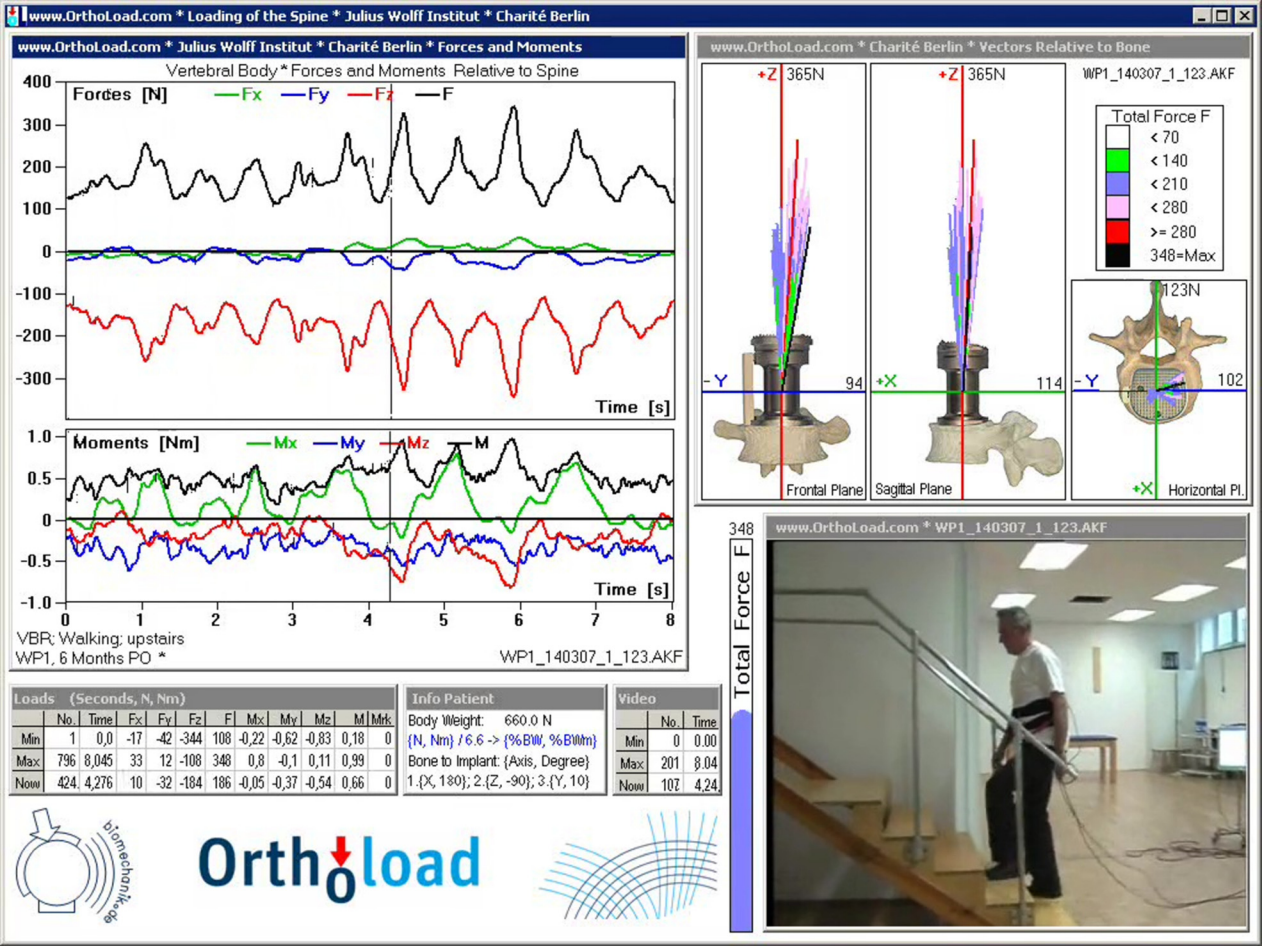
Load components and force vectors during walking up the stairs obtained from a telemeterized vertebral body replacement. Results from walking up five steps are shown. Videoclips of the original measurements with the synchronously shown loading data file wp1_140307_1_123 from database OrthoLoad.^17^ G. E. Bergmann, see http://www.OrthoLoad.com for “Charité Universitaetsmedizin Berlin, ‘OrthoLoad’ (2008)” (accessed April 1, 2021). Copyright 2008 Authors, licensed under a Creative Commons Attribution (CC BY) Unported License.

IVDs are composed of the nucleus pulposus (NP), the annulus fibrosus (AF), and the cartilaginous endplates (EP), which connect the IVD to the vertebral bodies. The composition of the NP and AF (Table I) supports the mechanical function of transferring and withstanding the loads that the spine is subjected to during daily activities. The IVD is subjected to constant complex loads as shown in Fig. 1. For example, a simple activity such as walking up the stairs will result in a complex load involving axial compression–tension (FZ), anterior–posterior shear (FX), lateral shear (FY), lateral bending (MX), flexion-extension (MY), and axial rotation (MZ) as shown in Fig. 2.^17^

**TABLE I.** NP and AF mechanical function and composition.^18^ Reproduced with permission from Molladavoodi *et al.*, Cell Tissue Res. **379**, 429–444 (2020). Copyright 2019 Springer-Verlag GmbH Germany, part of Springer Nature.

Characteristics	Nucleus pulposus (NP)	Annulus fibrosus (AF)
Mechanical function	Resisting compressive forces	Resisting tensile forces and containing NP bulging
Cells	Notochordal in early childhood gradually transform toward chondrocyte-like cells in the first decade of life	Fibroblasts toward the outer annulus and fibrochondrocytes toward the inner annulus
Extracellular matrix (ECM) structure and composition	Type II collagen network:	Concentric lamellae of alternating oblique collagen fibers interspersed with proteoglycans:
	Water (70%–90%), proteoglycans (50% of dry weight), type II collagen (20% of dry weight)	Water (60%–80%), proteoglycans (10%–20% of dry weight), collagen (50%–70% of dry weight), elastin (2%)

At a cellular level, these loading conditions have proven to play significant roles in the mechanisms involved in Degenerative Disc Disease (DDD). The cellular physiology is significantly affected by mechanical stimulation, demonstrating that mechano-biochemical factors are interdependent and can amplify each other.^6^ The magnitude and frequency of the load affect the IVD matrix strain and can perturb the integrin–ligand interactions, varying the cellular environment. This includes changing the pH, hydration levels and permeability,^19^ which will in turn affect the cell viability. Additionally, the IVD cell phenotype and behavior are also affected by mechanical stimuli.^20^ Changes in proteoglycan (PG) content, collagen expression, metalloproteinase activity, and matrix gene expression have been reported as a result of mechanical loading.^21–23^ Hyper- and hypo-physiological loading is detrimental to the disc health, causing trauma and leading to disc degeneration.^24^ A small or reduced load, e.g., immobilization, will result in reduced biosynthesis rates, while overloading can cause damage to the IVD structure.^25^ Static and dynamic overloading affect the IVD integrity negatively. For example, the posterior AF is mostly affected by static overloading, increasing the risk of posterior herniation, whereas the dynamic overloading results in cell death and matrix disruption throughout the IVD.^26^

The interdependencies between mechanical and biochemical factors suggest that mechanical tests simulating daily activities are imperative for the evaluation of regenerative approaches to treat DDD. These physiologically simulated models will allow us to better understand how loading affects the IVD on a cellular and tissue level when degeneration is present, and when therapies are introduced. The complexity of *in vivo* loading of the spine, involving a combination of forces and moments in six degrees of freedom, means that replicating such loads *in vitro* is a substantial challenge. However, doing so has the potential to provide more effective evaluation of regenerative therapies than simple loading protocols, or methods that omit loading completely.

### B. Considerations for IVD assessment models

**FIG. 3.**
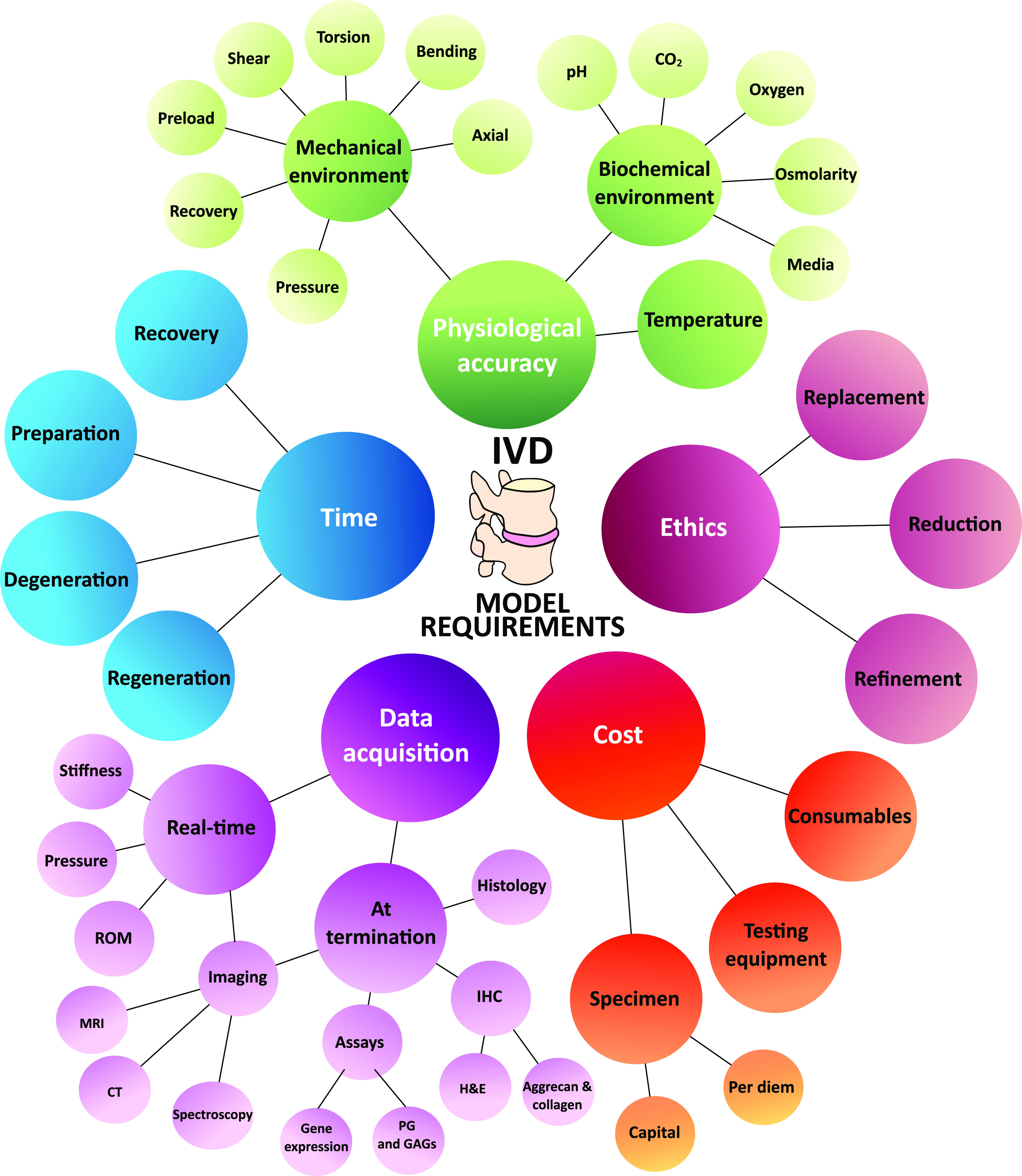
Requirements for IVD models which should be considered during experimental design.

**FIG. 4.**
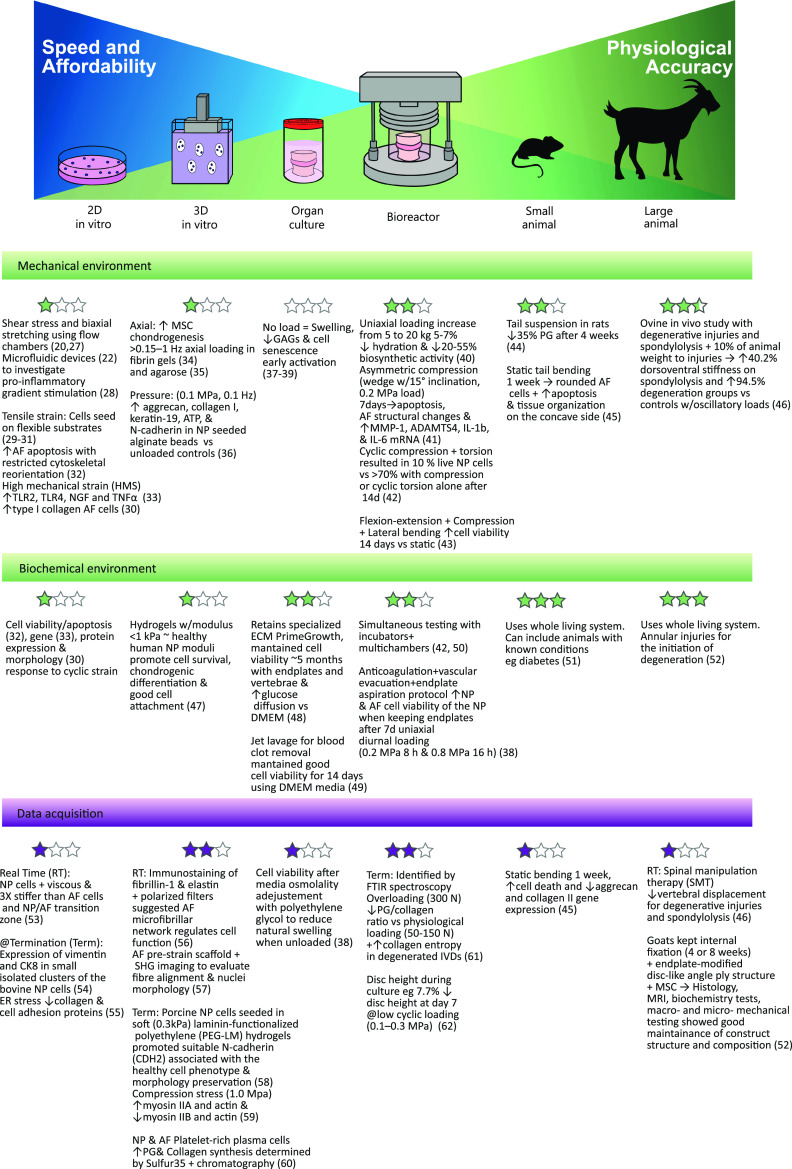
Model comparison based on physiological resemblance and data acquisition capabilities.

Current IVD models each offer their own advantages and limitations, all of which should be carefully considered during the framing of the problem and in the design of the experimental plan. A comprehensive set of selection criteria for the establishment of an ideal IVD experimental model are displayed in Fig. 3. This highlights the large variety of parameters that must be considered in relating an IVD model to the human *in vivo* IVD, as well as additional considerations relating to ethics, data acquisition, cost-effectiveness, and time considerations. Some examples illustrating the coverage of the ideal requirements for the study of the IVD are presented in Fig. 4.^20,22,27–62^

This current work presents a narrative review that aims to provide comprehensive coverage of the wide range of models currently in use that explore and exploit mechanical loading, and outlines key *in vivo*, *ex vivo*, and *in vitro IVD* mechanobiological studies. In this review, *in vitro* refers to cell studies, *ex vivo* is used for organ explant studies in which cells were maintained alive, and *in vivo* encompasses studies performed with live animals. The review builds in complexity and scale, discussing in sequence the current *in vitro*, *ex vivo*, and *in vivo* IVD models. First, the *in vitro* section presents the two-dimensional approaches that study the effect of shear stress and biaxial stretching on IVD cells, followed by the three-dimensional models relying on gels to increase the complexity of their IVD cell mechanical testing. Moving up in scale, the implementation of *ex vivo* whole organ models is discussed and compared with the simpler models. Two types of organ models have been included: a simple unloaded organ culture system to explain the capability and complexity of organ explant models and a whole organ model with mechanical loading using a bioreactor. Finally, small and large *in vivo* animal are described. The proposed selection criteria will be used to reflect upon the different IVD experimental models across the different scales. Strengths and limitations for all models are identified, compared and discussed, and areas of improvement and refinement are highlighted.

## II. *IN VITRO* MODELS

The effect of load on IVD cells has been studied *in vitro* using 2D and 3D models. The use of cell culture models allows, to a certain extent, the simplification of testing conditions but is limited in replicating the mechanical cues present *in vivo.* Current cell models are often tested without truly resembling the native (or diseased) microenvironment. For example, the substrates on which cells are seeded and tested do not reflect the behavior and characteristics of the human IVD extracellular matrix. Moreover, mechanical stimulation is often limited to a single axis, contrary to the six-axis loading to which IVDs are subjected.

*In vitro* models allow for simplified tests to understand the behavior of native IVD cells as well as the preliminary testing of cell-based therapies for the IVD. An example of this type of assessment is to understand how mesenchymal stem cells (MSCs) and novel progenitor cell populations within the IVD act under specific loading and environmental conditions.^63,64^ Having a deep understanding of these behaviors is necessary to tailor and develop better differentiation strategies that influence the desired cell phenotype and correct tissue formation.^65^ In addition, these *in vitro* models can provide valuable information to understand how stem cells survive and thrive in the IVD microenvironment accelerating the development of therapies.

### A. Two-dimensional cell models

When using conventional 2D cell models, mechanical loading is limited to applying shear stress and biaxial stretching. A range of approaches has been explored for assessing the impacts of shear stress on cell behaviors, including the use of plate flow chambers^20,27^ and microfluidic-based devices.^22,28^

**FIG. 5.**
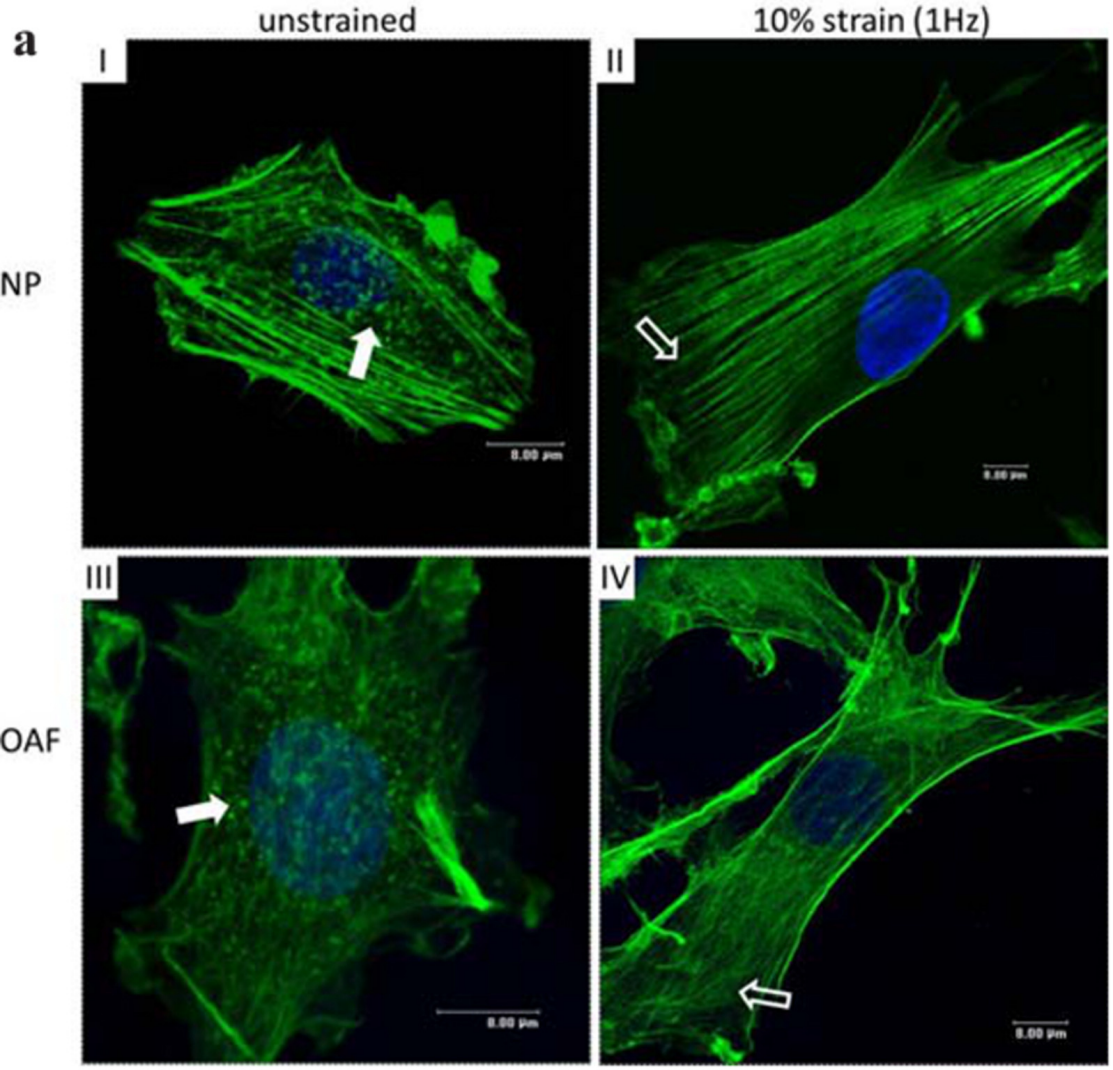
The effect of tensile strain on the actin cytoskeleton of intervertebral disc cells cultured on type I collagen. Visualization using Alexa-488™-phalloidin (scale bar = 8 *μ*m). F-actin organization in nucleus pulposus (NP) and outer annulus fibrosus (OAF) cells before and after 10% strain (60 min). Punctate F-actin labeling in NP and OAF cells (white arrows) were replaced with extensive F-actin stress fibers upon tensile strain.^30^ Reprinted with permission from Li *et al.*, Eur. Cells Mater. **21**, 508–522 (2011). Copyright 2011 Authors, licensed under a Creative Commons Attribution (CC BY) Unported License.

Numerous studies using IVD cells have also managed to integrate cyclic tensile strain during culture by seeding cells on flexible substrates.^29–31^ The use of cyclic strain has helped to understand cellular responses, including cell viability/apoptosis,^32^ gene^33^ and protein expression, and morphology.^30^ Essential variables to consider during the design of these models include the impact that the intrinsic properties (such as stiffness or viscoelasticity) of the substrate on which the cells are cultured may have on cell behavior, regardless of the applied stimuli.^66,67^ The perfect matching of material and IVD tissue represents a challenge, as the IVD is viscoelastic, and possesses significant zonal variations, in addition to altered behavior once degenerated. The consideration of the matrix creep or stress relaxation is essential to understand the IVD cellular interactions with their native microenvironment and to predict their response to different biomaterials.^68^ For example, when bovine IVD cells seeded in type I collagen-coated culture plates underwent a 10% strain elongation for 60 min, re-organization of F-actin in NP and outer AF cells was observed,^30^ as shown in Fig. 5. These changes were not organizational as they resulted in a 1.5–2 fold increase in the β-actin transcription in the NP and AF cells.

### B. Three-dimensional cell models

Three-dimensional (3D) cell models, which rely on support systems such as hydrogels,^69^ may provide an ability to replicate the viscoelasticity and effects of the extracellular matrix of the IVD, which is not possible in 2D cell models. The interaction of IVD cells and the environment also regulates and affects gene expression and other cell functions.^70^ The effect of loading in different cell populations has been investigated using this type of model. The impact of dynamic pressure on notochordal (NC) and mature nucleus pulposus (MNP) cells using alginate beads, under both low (0.4–0.8 MPa) and high (1.6–2.4 MPa) stress conditions for a 24-h period has shown that NC cells have higher resilience toward acute mechanical stress compared to MNP cells.^71^ The phenotypic expression and mechanobiology of NP cells have also been shown to be pressure magnitude dependent.^36^

The use of 3D cell models allows more flexibility in terms of mechanical loading; however, the substrates and gels for these models must be carefully considered as they affect the cell shape, mitosis and ECM production.^18^ The environment in which the cells are seeded will affect the way the loading is perceived by the cells and is therefore a critical aspect for the development of 3D cell models, and the selection of potential regenerative materials. The cell density within the 3D structure has also been shown to play a crucial role in the mechanobiological response of NP cells to hydrostatic pressure^36^ suggesting that further optimization of culture conditions it is required when considering tissue engineering approaches for the treatment of the NP.

## III. *EX VIVO* MODELS

Explant organ models include culturing the organ in cell culture flasks, biochambers, and microfluidic devices. The culture of whole IVDs has been used to study IVD degeneration. This provides a means to investigate the effect of different experimental parameters on individual cells or tissues of the IVD, and also provide an understanding of how this relates to the IVD structure as a whole. These models generally use samples from animals widely available in local abattoirs, such as bovine tail IVDs, making them easily accessible.

### A. Organ culture

Human and animal (e.g., bovine and rat) whole IVDs cultured in flasks and biochambers have been used for biochemical tests, histology, and gene expression.^48,72–74^ Microfluidic devices have been optimized for small IVD samples, for example, IVDs extracted from mice, but the implementation of mechanical testing has been limited.^75^ Microfluidics allows the maintenance of a nurturing environment and maintains good cell viability for longer than static organ culture.^75^

**FIG. 6.**
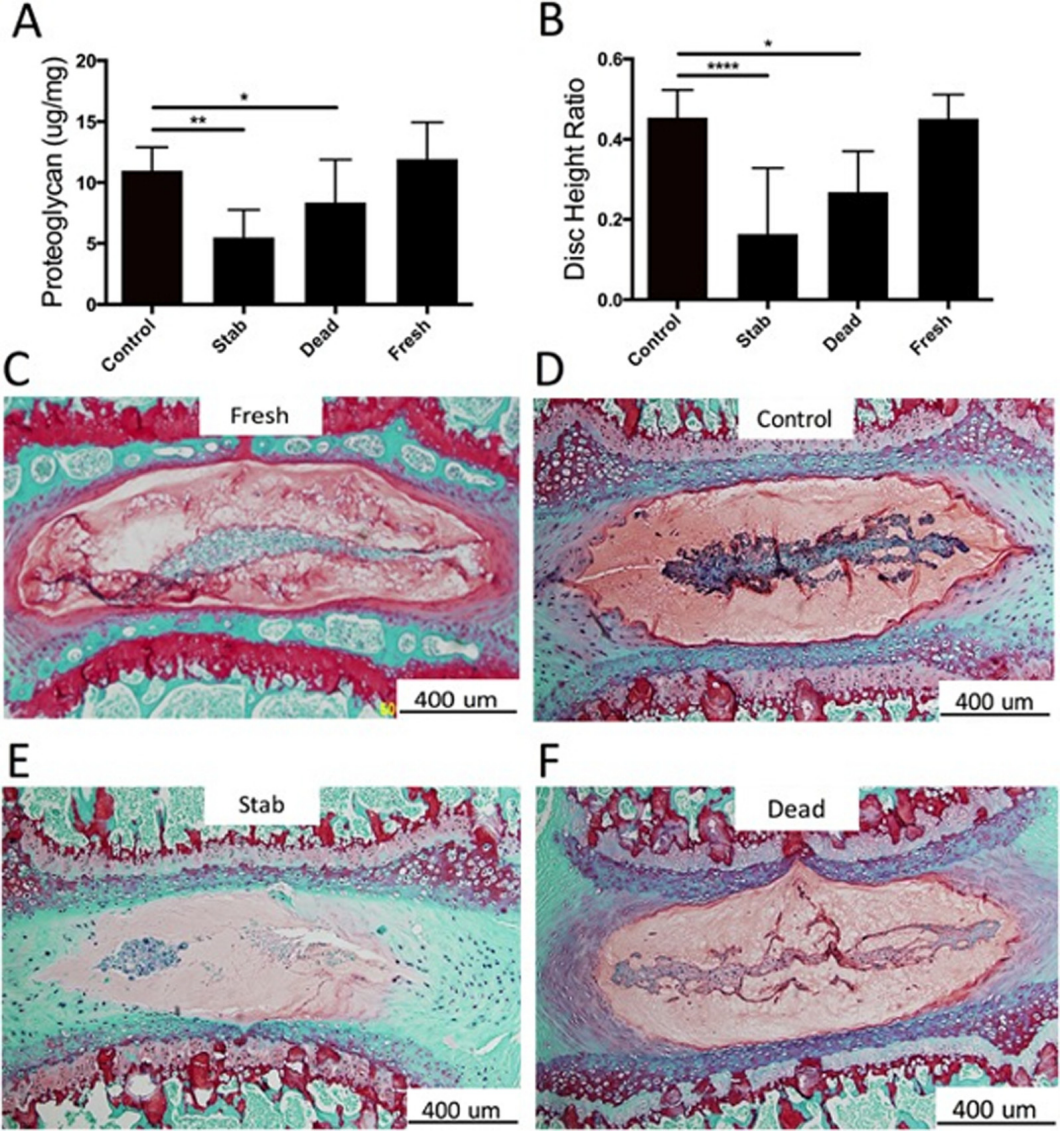
Structure and composition of fresh, death and culture IVDs (control and after injury). (a) GAGs quantification; (b) disc height in all groups; (c)–(f) Safranin‐O staining in all disc. Reprinted with permission from Abraham *et al.*, J. Orthop. Res. **34**(8), 1431–1438 (2016). Copyright 2016 John Wiley and Sons.^78^

Whole organ models allow for the creation of degenerative models through accelerated effects of ageing and external trauma. For example, the injection of enzymes that degrade the ECM can be used on these models,^76,77^ presenting evidence of degeneration from day 3.^74^ Abraham *et al.* used a murine whole organ model to demonstrate the degeneration caused by a stab injury after culturing for 21 days, while comparing the degeneration with a cultured non-injured control (Control), a cultured flash frozen (Dead) disc, and a fresh (day 0) non-cultured (Fresh) IVD^78^ as shown in Fig. 6.

Daily activities involve dynamic loading and can be significant contributors to slow degenerative processes when overloading or hypomobility is involved.^79^ Furthermore, the use of physiological loads have been shown to preserve matrix function.^80^ The need for improvement in the physiological resemblance has led to the development of *ex vivo* whole organ models that also integrate mechanical loading to the IVD.

### B. Bioreactors

When new therapies are evaluated, the incorporation of dynamic mechanical testing is necessary to predict the behavior of the proposed material under physiological and daily activity conditions.^81^ For the purposes of this review, a bioreactor is defined as a whole organ IVD culture model that has been adapted to provide the additional capability to apply non-static mechanical loading to the IVD during the culture period, leading to a more physiologically relevant IVD testing model.^38,40,43,62,82^

Bioreactors have been employed in similar investigations as organ culture models, through the use of enzymatic degeneration and mechanical injury protocols.^83^ However, bioreactors provide the additional capability to investigate the effects of overloading or how different loading protocols affect the IVD, as well as offering the ability to complete real-time biomechanical data acquisition. Although the integration of loading in organ culture represents a step toward improving the resemblance of the human physiology in IVD models, complex loading in bioreactors remains limited.

**FIG. 7.**
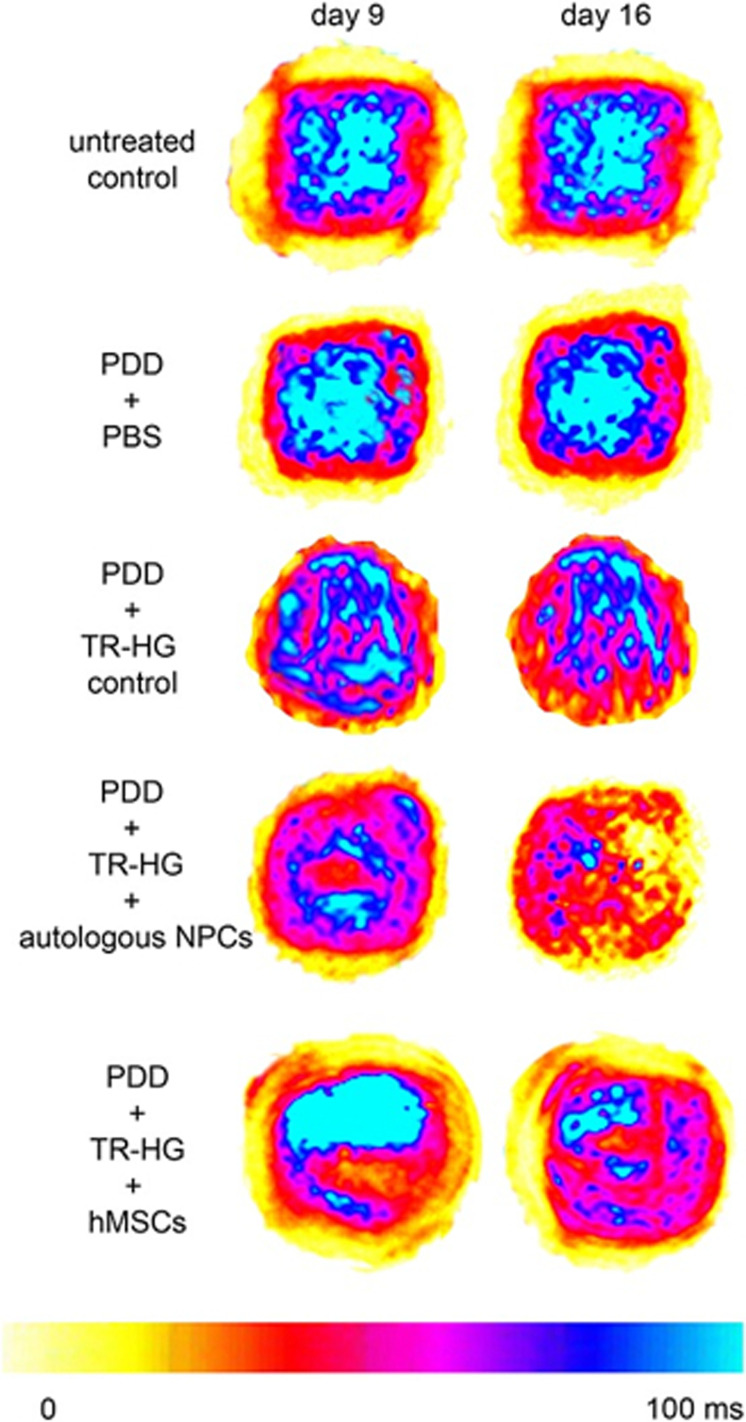
MRI T2^*^ mapping of transverse sections of bovine discs at day 9 (before loading) and day 16 (after loading). The blue scale represents higher content of water while the orange-yellow indicate lower water content. PDD represents papain disc digestion. The untreated control has been injected with Phosphate Buffered Saline (PBS); PDD + PBS injection; PDD + thermo-hydrogel (TR‐HG, material control); PDD + TR‐HG + autologous bovine NP cells (bNPCs); and PDD + TR‐HG + hMSCs^86^ Reproduced with permission from Malonzo *et al.*, J. Tissue Eng. Regener. Med. **9**(12), E167–E176 (2013). Copyright 2013, John Wiley & Sons, Ltd.

Bioreactors offer the possibility of acquiring data frequently captured during biomechanical studies of the spine, such as stiffness, range of motion, and measurements relating to the neutral zone.^84,85^ Live data acquisition throughout a culture period provides a significant advantage over other models. This information can be used to evaluate the effect of degenerative initiators or the stability and efficacy of regenerative constructs. Where real-time biomechanical data acquisition is not possible, such evaluation can be completed after testing, similar to *in vivo* models. For example, Malonzo *et al.* evaluated the effect of static loading on a papain‐induced bovine caudal disc degeneration model (PDDM) treated with thermo-reversible injectable cell‐embedded hydrogels. This group used MRI T2^*^ mapping before and after loading, as shown in Fig. 7, identifying the gels' compression and the collapsed disc space after loading.^86^

The application of load is a key advantage of the bioreactors. When testing protocols are designed, the type of loading, magnitude, frequency, number and type of cycles, and preload should be carefully selected as the cell behavior, gene expression, and mechanical response vary depending on the loading conditions.

**FIG. 8.**
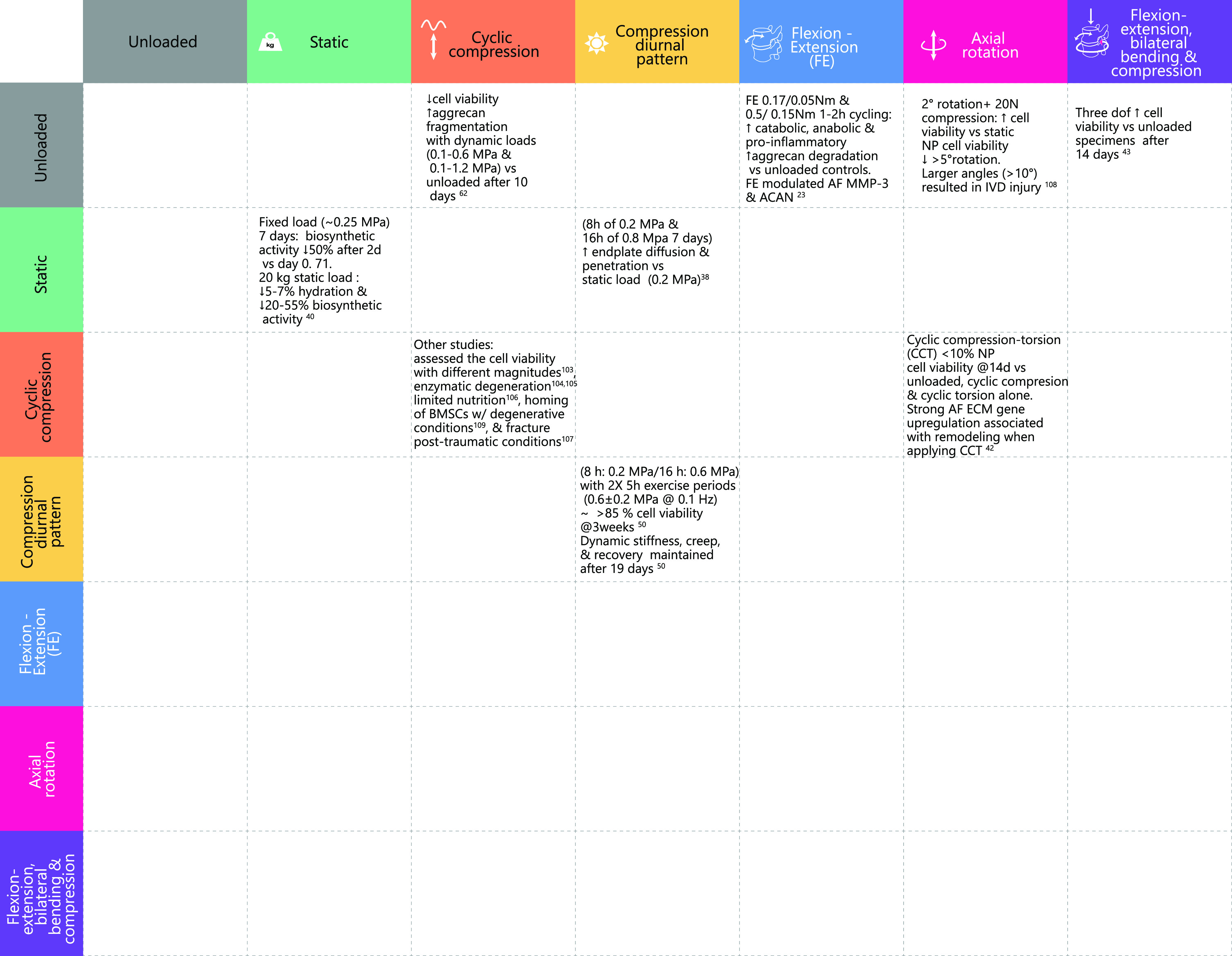
Load capability and comparison of bioreactor studies.

Mechanical loading has a strong correlation with several biological effects such as metabolic and structural response.^79^ Multi-axis or complex loading protocols have been used widely to investigate the biomechanics of functional spinal units or non-cultured IVD specimens.^84,87–96^ However, the application of multi-axis loading to a bioreactor system has been more limited in terms of the loading conditions. The majority of loading IVDs have been subjected to in *ex vivo* whole-organ models is uniaxial compression,^38,39,62,97–100^ though axial compression has been combined with axial torsion,^42^ and preliminary research has been completed to apply a combination of bending moments to *ex vivo* IVD specimens^43^ as shown in Fig. 8. However, to fully replicate the biomechanical environment, it is critical to apply adequate axial compressive loading, as this alters the segmental motion patterns, neutral zone, and stiffness compared to bending without such a preload.^23,38,40,42,43,50,62,101–109^

Developing and implementing multi-axis systems for IVD and spine testing is extremely challenging due to the complex interactions and interdependencies between the different axes, combined with the non-linear material properties of the tissues of the IVD.^102^ Dynamic testing rates can lead to errors and delays in coordination, resulting in unstable or unpredictable behavior hindering the ability to replicate physiological loading accurately. Position, load, and hybrid control methods are three common approaches used in control systems engineering and have been employed to regulate the behavior of spine test systems. Full load control presents a greater challenge to apply to specimens, as the initial stiffness and flexibility of a sample are unknown, but it provides the opportunity to use available load data from *in vivo* human studies.^146^ Position control enables greater consistency, minimizing viscoelastic effects and artifact forces and moments. However, there is limited human *in vivo* kinematic data of the spine in six degrees of freedom, which limits the ability to replicate the kinematics of the spine during normal daily activities using a position control *in vitro*. The integration of physiological loads matching values presented during human activities in several axis (ideally six-axis) could provide relevant data to understand the mechanical behavior of the human IVD and evaluate the biochemical changes under specific conditions. Evaluation of human-like parameters is necessary to translate the findings of *in vitro* and *ex vivo* studies into animal testing and clinical settings.

## IV. *IN VIVO* MODELS

Human *in vivo* studies provide the most accurate way to evaluate the safety and efficacy of treatments, but require substantial pre-clinical evidence prior to being undertaken, in order to minimize risks to patients. Even though, human *in vivo* testing is the most relevant model; data acquisition is limited compared to *in vitro* and *ex vivo* models or animal *in vivo* studies due to the *ex vivo* sample processing or invasive procedures needed for specific tests. Furthermore, these studies usually are long as they require ethical approval, and extended periods for patient recruitment and follow-up.^110^ In addition, the status of the patients' disc health must be assessed. In studies in which several patient visits are required, enrollment dropout is a possibility that can impact the cost and time to final results.^111^

Therefore, animal models provide a valuable step in the research and development process prior to human *in vivo* testing. Animal *in vivo* models that have been integrated into spine research have included caprine,^112^ rat,^113^ mouse,^114^ canine,^115^ ovine,^46^ ape,^116^ and rabbit^117^ models. The use of *in vivo* animal studies offers high physiological accuracy by allowing the application of mechanical stimuli while maintaining the natural environment and active living systems.

Surgically induced *in vivo* models of degeneration represent a challenge, as while they are good for the introduction of an injury, which may trigger a degenerative cascade, they do not generally replicate the complex nature of the disc degeneration.^44^ Among the reported studies, tail suspension,^118^ axial loading,^119^ torsional injury,^120^ lumbar fusion,^121^ annular damage,^122^ and endplate injury^123^ have been reported using *in vivo* animal studies. In these studies, the effect on the natural IVD microenvironment can be investigated as well as allowing the whole living system to react to that specific injury. These significant advantages make *in vivo* studies the most physiological accurate models for the study of DDD and regenerative therapies. On the other hand, different species will vary in cell environment, mechanics, geometry, and size. These differences have been extensively discussed^37,44,124–128^ and should be considered when selecting an animal model. For example, unlike humans, the NP of pigs, rabbits, and non-chondrodystrophoid dogs maintain a high level of notochordal cells throughout their lifetime, which is associated with the absence of intervertebral disc degeneration (IVDD) in these animals under normal conditions.^129^

**FIG. 9.**
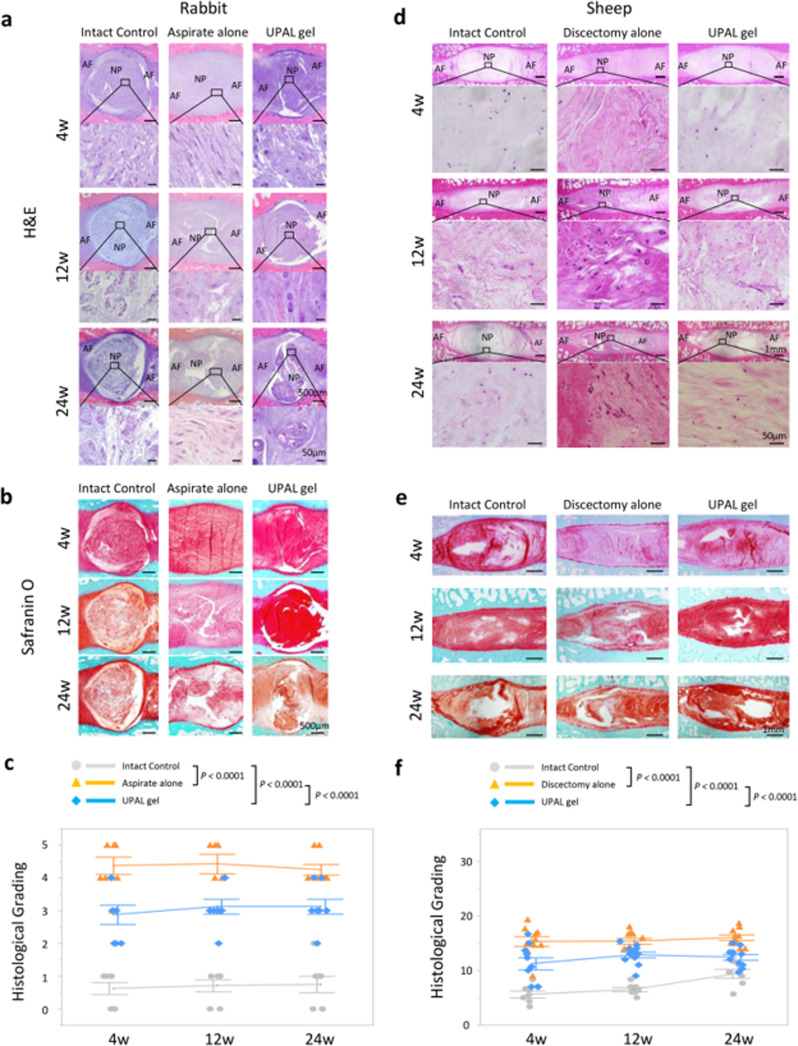
Midsagittal sections of rabbit intervertebral discs (IVDs). UPAL, ultra-purified alginate. AF; annulus fibrosus, and NP; nucleus pulposus. (a) Hematoxylin and Eosin (H&E); (b) safranin O. (c) Histological grading in rabbit discs. Midsagittal sections of sheep IVDs stained (d) H&E; (e) safranin O; and (f) Histological grading in sheep discs.^131^ Reproduced with permission from Tsujimoto *et al.*, EBioMedicine **37**, 521–534 (2018). Copyright 2018 Authors, licensed under a Creative Commons Attribution (CC BY) Unported License.

Many *in* vivo studies do not restrict the animal movement or attach invasive instruments such as rigs or jigs. Such rigs or jigs have included the manipulation of bone screws/pins to alter the position or load applied to the animal vertebrae to more accurately understand the effects of specific loads^130^ but these may not represent the same loads encountered during daily activities such as walking. Furthermore, the risk of infection on open wounds and the correct positioning of mechanical test fixtures is a challenge. Due to the limited tissue area on small species, it is easier to work and manipulate larger animal models but adapting mechanical devices while controlling testing conditions becomes a greater challenge. Tsujimoto *et al.* integrated a dual approach using a rabbit and a sheep model for the proof-of-concept for an acellular bioresorbable ultra-purified alginate gel (UPAL) for IVD repair as shown in Fig. 9. In this study, AF punctures were used to create defects in rabbit and discectomy in sheep, followed by the injection of the UPAL gel and a gelation solution. The animals were sacrificed after 4, 12, and 24 weeks and histological grading was performed, which suggested that the regenerative treatment prevented IVD degeneration when compared with the discectomy alone.^131^ By integrating a dual approach (small and large animal model), the pre-clinical proof-of-concept stage of this gel therapy was completed and met regulatory requirements to move into its first-in-human clinical trial (Hokkaido University Hospital approval number: H30–10 and Eniwa Hospital approval number: dMD001-H1).^132^

Small animal *in vivo* studies can produce results in approximately 3 weeks.^133^ *In vivo* models that adopt surgical degeneration procedures and require post-surgery recovery and follow-up require longer, and can last from 3 to 12 months when using large animals.^134^ These extended recovery periods can represent an advantage over *ex vivo* studies, allowing a wider observation window to evaluate potential healing and recovery processes. However, recovery time after surgery results in additional expenses, which also should be considered. In studies in which real-time data acquisition is intended, the incorporation of sensors, such as percutaneous transducers, needles, and piezoresistive sensors has been explored. Such sensors can be placed in the IVD to measure pressure, and studies adopting these techniques have been used to collect *in vivo* data in the immediate period following surgery, which allows the testing protocols and animal euthanization to be completed within 24 h.^135–137^

Creative arrangements involving loading frames, actuators, and supports to stabilize the animal trunk are usually needed when applying loads in large animal *in vivo* studies. Most published studies have relied on applying controlled loading. A small number of these studies have evaluated the mechanical response of the IVD while the animal was allowed to carry out non-controlled activities.^136,138^ The use of sensors for localized *in vivo* measurements offer an excellent alternative to obtain data during regular activities^139^ and may have fewer ethical implications compared to the use of invasive loading fixtures. Nonetheless, design parameters, such as geometry and size, will affect the measurements. Therefore, the data obtained from these devices should be carefully evaluated in order to allow translation across *in vitro*, *ex vivo*, and *in vivo* models.

## V. DISCUSSION

This review has aimed to identify the models currently used for the study of the intervertebral disc, degenerative disc diseases, and regenerative treatments. These models include *in vitro*, *ex vivo*, and *in vivo* models. The *in vitro* models comprise 2D and 3D cell cultures, and *ex vivo* models include whole IVD organ cultures and whole IVD bioreactors. *In vivo* models can be subcategorized based on the animal size. Small animal models rely on the use of mice, rats, and rabbits, whereas large animal models are generally limited to the use of goats and sheep. This review has presented the advantages and limitations of each model based on time, cost, ethics, physiological relevance, and data acquisition.

**FIG. 10.**
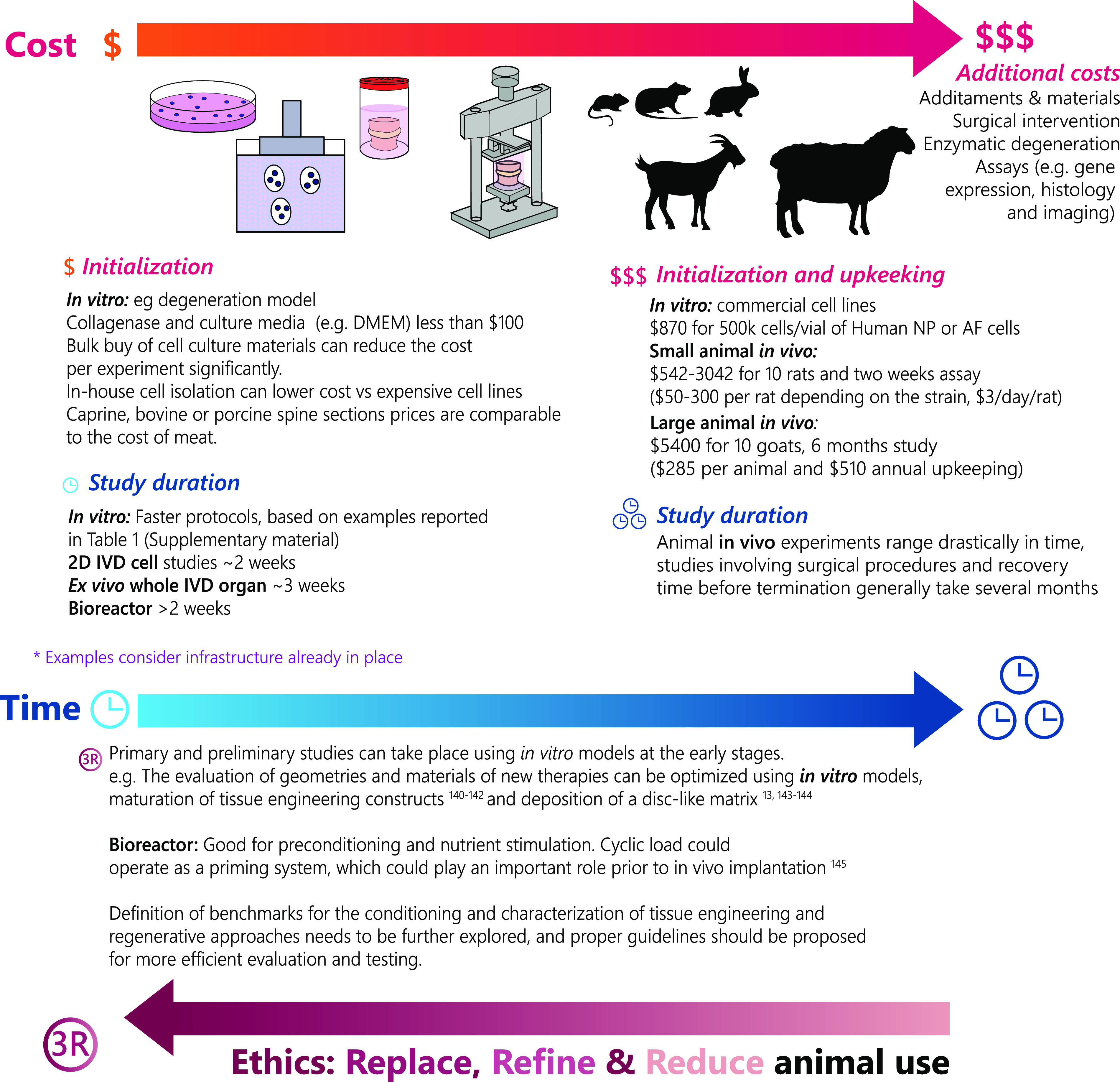
Cost, time and ethics implication of *in vitro* and *in vivo* IVD models. The cost of animals was estimated using catalogues from companies selling cell lines,^146^ research models (e.g., Charles River 2020 Catalogue^147^) and animal husbandry markets and prices reports in the UK.^148^ Prices for animal upkeeping were acquired from internet sources (e.g., costs of Raising Goats in 2019^149^) and anecdotal information from colleagues working with small and large models in the USA and Europe. The estimated expenses present a rough estimate and might vary in different geographies and be impacted by bulk purchases and infrastructure in place.

**FIG. 11.**
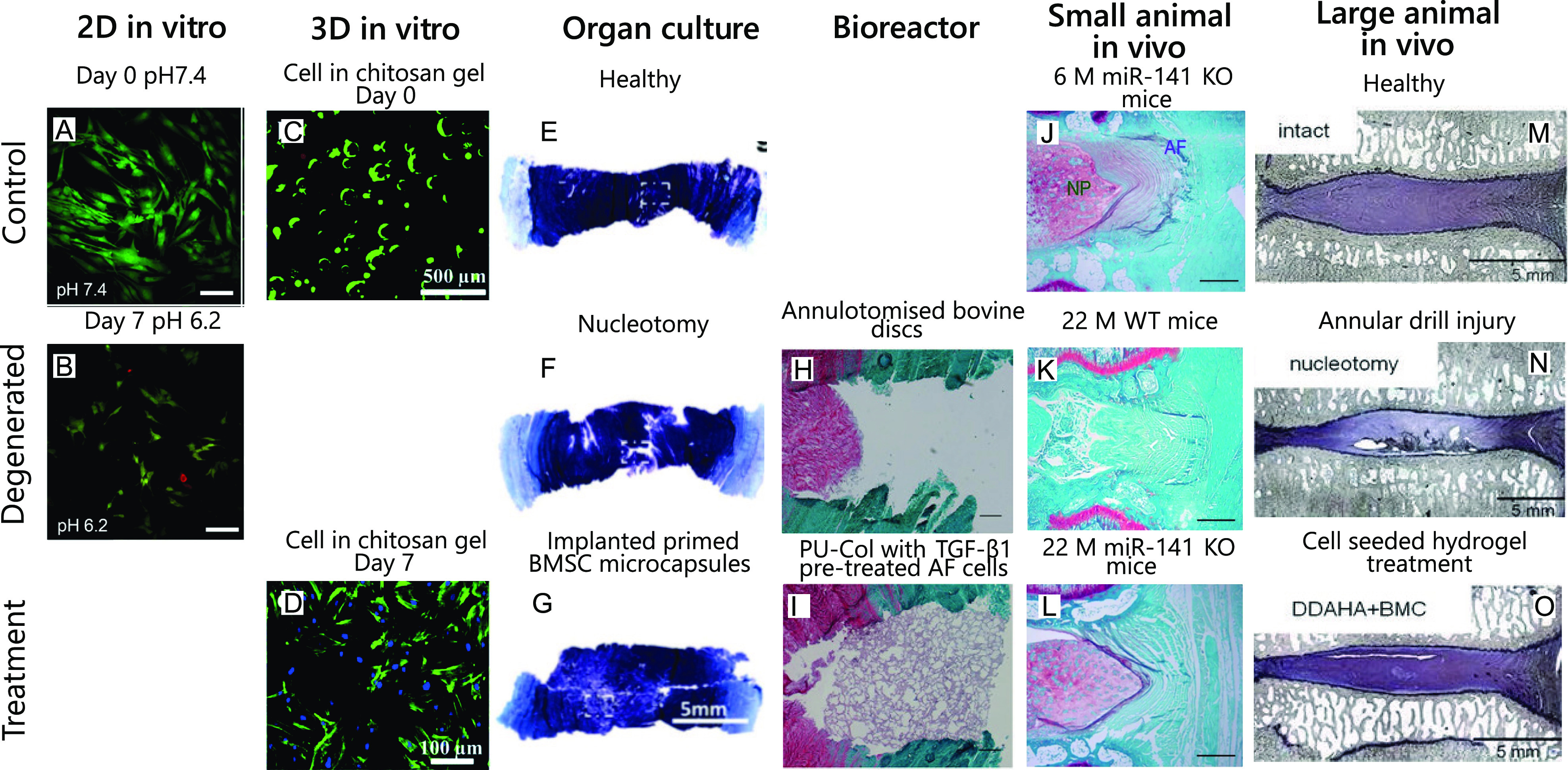
Examples of studies using the different approaches to investigate the degeneration and treatment of the IVD. (a) and (b) NP cells cultured at different pH at day 0 and day 7. Green cells represent live cells, while red cells represent dead cells. Non-degenerate environment was represented by pH 7.4 while a severely degenerated environment was simulated with pH 6.2.^150^ Reproduced with permission from Gilbert *et al.*, Sci. Rep. **6**, 28038 (2016). Copyright 2016 Authors, licensed under a Creative Commons Attribution (CC BY) Unported License. (c) and (d) Live/dead (green/red) staining of IVD cells seeded on chitosan hydrogel for 24 h and 3 D [cytoskeleton filaments (green), nuclei (blue)].^151^ Reproduced with permission from Yang *et al.*, RSC Adv. **8**, 68 (2018). Copyright 2018 Authors, licensed under a Creative Commons Attribution (CC BY) Unported License. (e)–(g) Histological evaluation of *ex vivo* cultures, stained with aldehyde fuchsin and alcian blue to identify sGAG and deep purple to indicate GAG accumulation. Images shown represent a healthy disc, a disc with nucleus injury and primed microencapsulated bone marrow stromal cells (BMSCs) (all after 28 days of culture).^152^ Reproduced with permission from Naqvi *et al.*, Eur. Cells Mater. **37**, 134–152 (2019). Copyright 2019 Authors, licensed under a Creative Commons Attribution (CC BY) Unported License. (h)–(i) Safranin O/fast green-stained IVD sections after 15 D organ culture including annulotomised discs and annulotomised discs repaired with polyurethane and collagen scaffold seeded with TGF-β1-pre-treated AF cells (PU-Col-AFCs) and compressed for 1 h at 0.02–0.2 MPa, 0.2 Hz daily.^153^ Reproduced with permission from Du *et al.*, Eur. Cells Mater. **39**, 1–17 (2020). Copyright 2020 Authors, licensed under a Creative Commons Attribution (CC BY) Unported License. (j)–(l) Genetic deletion of miR-141 suppresses spontaneous and surgically induced IDD. Safranin O staining of intervertebral disc from 6-month-old mice and 22 month-old mice with and without gene deletion (WT: wild-type).^154^ Reproduced with permission from Ji *et al.*, Nat. Commun. **9**, 5051 (2018). Copyright 2018 Authors, licensed under a Creative Commons Attribution (CC BY) Unported License. (m)–(o) Mid-sagittal histological sections of the disc intact, with nucleus injury and with treatment composed of dodecyl-amide of hyaluronic acid (DDAHA) hydrogel and bone marrow derived mononuclear cells (BMC) after 12 weeks.^155^ Reproduced with permission from Reitmaier *et al.*, Eur. Spine J. **23**, 19–26 (2014). Copyright 2014 Authors, licensed under a Creative Commons Attribution (CC BY) Unported License.

Cost, time, and physiological accuracy are the core of experimental design in IVD research. *In vitro* models offer speed and affordability, when infrastructure is in place, while *in vivo* models may provide greater physiological accuracy. The differences in cost of *in vitro* and *in vivo* models vary significantly as shown in Fig. 10, which highlight the differences in cost for the initialization of *in vitro* and *in vivo* models.^13,140–149^ Figure 11 illustrate the use of the different models to investigate the degeneration and possible treatment of the IVD. A series of examples representative of the discussed models has been presented in Table I (supplementary material). This table allows us to compare the complexity of each model regarding inputs, outputs, experimental design, mechanical loading, and data acquisition.^150–155^

The complex, multi-axis loading that the spine is subject to during daily activities is well-reported; however, the integration of different degrees of freedom replicating everyday activities has been limited in organ studies using a bioreactor. One of the greatest advantages of *in vitro* models is the replicability and parallel testing of specimens and cells, resulting in considerable data collection while reducing experiment times and costs. Bioreactors are equipped with individual chambers which can maintain an individual disc. Some research groups have implemented multi-chamber reactors, but the capacity for parallel analysis is still limited. This situation should be considered as another development area to exploit the full potential of this approach. Parallel complex *in vivo* loading in multiple whole intervertebral discs while replicating an *in vivo* environment through controlled cell culture conditions could offer an affordable, fast, ethical, and highly physiologically accurate model for IVD related studies.

Moreover, there is scope to develop *in vivo* models to provide greater data from fewer animals, and scope for the refinement of *in vivo* models of degeneration to better reflect different levels of degeneration and to better understand the effectiveness of regenerative treatments. The use of quantifiable and faster degenerative methods would also be beneficial to reduce the time taken to complete *in vivo* animal studies, which would not only improve the efficiency of the research but also reduce any animal suffering that may occur. Animal selection should be strictly justified during experimental design and ethical approval applications. However, there is no perfect animal model which can replicate human disc degeneration. Nevertheless, based on IVD geometry, and allowing scalability with dimension dependent characteristics, such as disc pressure, large animal models, are much more suitable for the translation to the human IVD. If rapid and characterizable degeneration can be induced in large animals, the need for testing using in small animals might be reduced.

When working with animals, there is less control over how a specific load can be applied in comparison with the *in vitro* approaches. This issue represents a limitation on the possible inputs on *in vivo* studies. Small *in vivo* animal studies allow for easier mechanical manipulation. In the same way, small animals such as rats and mice can be encouraged after degenerative procedures to carry standardized activity such as running and standing. These opportunities offer limited useful information based on the anatomical differences when compared to humans. Large animal models could provide this information, but the lack of standardized protocols for regular activities in large animals represents a challenge for comparison between studies, or for translation to the human IVD. In the same way, repeatability of activities cannot be easily achieved with large species. It is possible that output parameters may be evaluated more successfully in *ex vivo* models, if the application of mechanical loading can be improved to accurately simulate complex physiological loading.

Genetic inheritance, age, inadequate metabolite transport, and loading history have been identified as causes of the weakening of discs which eventually can lead to structural failure and pain.^156^ Overloading has been widely reported as a cause for degeneration, while physical inactivity has a strong correlation with disc degeneration at the thoracic and lumbar levels.^26,157–159^ Loading is essential to maintain the disc in healthy conditions. Certain types of loading have been reported to slow down degeneration,^160^ while rat studies have shown that exercise increases cell populations in the IVD and reduces pain.^161,162^ A historical cohort study showed that in later adulthood back pain was less common in former athletes when compared with a control population. When sports were compared, signs of degeneration were presented in football players, and power sports athletes.^163^ Running has been proposed as an exercise regimen which can help to strengthen the disc. A study comparing different running patterns established that better hydration and glycosaminoglycan levels were shown in runners compared to non-athletic subjects. In this same study, hypertrophy in long-distance running was identified in the IVD in contrast with the jogging group.^164^ These comparisons confirm that the loading characteristics and conditions are critical for maintaining the disc or leading to injury and degeneration. Understanding and investigating different types of loading should remain an ongoing focus in IVD research, as this information can be integrated into the current protocols to evaluate DDD, regenerative approaches, and refine the exercise guidelines for pain management and prevention.

Live data acquisition during mechanical testing provides valuable information regarding the IVD and spine response to loading. The gathering of data during and after testing is ideal for comparison and to evaluate the IVD response under different conditions and time points. Imaging methods can be used on cell culture, before and after fixation, which allows for chemical and behavior characterization. IVD adaptations in stiffness and pressure are examples of parameters that can be assessed live when using *ex vivo* organ cultures and *in vivo* models. Although highly relevant, live data collection during *in vivo* testing is not often performed. The capability of the models might not allow for data collection, and equally, the possible parameters for data collection, might not be relevant for the scope of the studies. Similarly, the incorporation of data collection during experimentation with animals *in vivo* might be restricted due to the potential to induce pain and stress, or it may limit other aspects of the study if incorporated. The use of imaging during live testing is currently limited, but the use of sensors and telemetry may offer a suitable alternative to improve live data acquisition during activity and loading. However, technology and protocols should be carefully implemented, and the deployment cost should be considered.

## VI. CONCLUSION

*In vitro*, *ex vivo*, and *in vivo* models are part of the testing strategies for the study of DDD and associated regenerative approaches. So far, loading history, environmental factors, genetics, and age have been identified as factors contributing toward the degeneration of the disc. Appropriate model selection is a vital aspect of experimental design, and physiological resemblance and data acquisition must be taken into consideration.

*In vitro* models are effective for the study of isolated cellular processes, *ex vivo* whole disc studies have allowed integrating loading, while *in vivo* studies offer the best physiological accuracy. A general aim in the investigation of DDD and regenerative treatments is to simulate the *in vivo* environment accurately. However, the use of animal models, and in particular, large animal models generally require more time than cell and organ scale models, and may be considered unethical if these models can provide a valid alternative, particularly at the early stages of basic research or device development.

The integration of more realistic loading conditions to whole organ *ex vivo* disc studies using a bioreactor could help to bridge the gap between *in vitro* and *in vivo* models. *In vivo*-like loading using bioreactors could offer greater control over test conditions. This improvement could result in a reduction and even the replacement of animals in research at different stages of device development. Large animal models are the gold standard in pre-clinical testing of disc medical devices, and although this model is likely to remain as a requirement before human trials, further improvement of *in vitro* and *ex vivo* models to replicate more of the mechano-biochemical environment is expected to be highly impactful in future IVD research endeavors.

## SUPPLEMENTARY MATERIAL

See the supplementary material for details: a series of studies that integrated mechanical testing as part of their scope across all scales, *in vitro* (2D and 3D), bioreactors, and in vivo (small and large animal), have been summarized and presented in Table I in the supplementary material. This table facilitates the comparison of complexity of each model regarding inputs, outputs, experimental design, mechanical loading, and data acquisition.

## Data Availability

Data sharing is not applicable to this article as no new data were created or analyzed in this study.
